# Curcumin Enhances the Antitumoral Effect Induced by the Recombinant Vaccinia Neu Vaccine (rV-*neu*T) in Mice with Transplanted Salivary Gland Carcinoma Cells

**DOI:** 10.3390/nu12051417

**Published:** 2020-05-14

**Authors:** Chiara Focaccetti, Monica Benvenuto, Sara Ciuffa, Sara Fazi, Manuel Scimeca, Alessandra Nardi, Martino Tony Miele, Andrea Battisti, Elena Bonanno, Andrea Modesti, Laura Masuelli, Roberto Bei

**Affiliations:** 1 Department of Human Science and Promotion of the Quality of Life, San Raffaele University Rome, Via di Val Cannuta 247, 00166 Rome, Italy; chiara.focaccetti@uniroma5.it (C.F.); manuel.scimeca@uniroma2.it (M.S.); 2 Department of Clinical Sciences and Translational Medicine, University of Rome “Tor Vergata”, Via Montpellier 1, 00133 Rome, Italy; monica.benvenuto@unicamillus.org (M.B.); saramhh@hotmail.it (S.C.); modesti@med.uniroma2.it (A.M.); 3 Saint Camillus International University of Health and Medical Sciences, via di Sant’Alessandro 8, 00131 Rome, Italy; 4 Department of Experimental Medicine, “Sapienza” University of Rome, Viale Regina Elena 324, 00161 Rome, Italy; sarafazi@hotmail.it (S.F.); laura.masuelli@uniroma1.it (L.M.); 5 Department of Biomedicine and Prevention, University of Rome “Tor Vergata”, Via Montpellier 1, 00133 Rome, Italy; elena.bonanno@uniroma2.it; 6 Fondazione Umberto Veronesi (FUV), Piazza Velasca 5, 20122 Milano, Italy; 7 Department of Mathematics, University of Rome “Tor Vergata”, Via della Ricerca Scientifica 1, 00133 Rome, Italy; alenardi@axp.mat.uniroma2.it; 8 Department of Experimental Medicine, University of Rome Tor Vergata, Via Montpellier 1, 00133 Rome, Italy; miele@med.uniroma2.it; 9 Maxillo Facial Oncologic and Reconstructive Unit, “Sapienza” University of Rome, Policlinico Umberto I, 00161 Rome, Italy; andrea-batti@libero.it; 10 Neuromed Group, ‘Diagnostica Medica’ & ‘Villa dei Platani’, 83100 Avellino, Italy

**Keywords:** vaccine, cancer, ErbB2/Neu, curcumin, head and neck, immune response, tumor infiltrating leukocytes

## Abstract

The survival rate for head and neck cancer patients has not substantially changed in the last two decades. We previously showed that two rV-*neu*T intratumoral injections induced an efficient antitumor response and rejection of transplanted Neu (rat *ErbB2/neu* oncogene-encoded protein)-overexpressing salivary gland tumor cells in BALB-*neu*T mice (BALB/c mice transgenic for the rat *ErbB2/neu* oncogene). However, reiterated poxviral vaccinations increase neutralizing antibodies to viral proteins in humans that prevent immune response against the recombinant antigen expressed by the virus. Curcumin (CUR) is a polyphenol with antineoplastic and immunomodulatory properties. The aim of this study was to employ CUR administration to boost the anti-Neu immune response and anticancer activity induced by one rV-*neu*T intratumoral vaccination in BALB-*neu*T mice. Here, we demonstrated that the combined rV-n*eu*T+CUR treatment was more effective at reducing tumor growth and increasing mouse survival, anti-Neu humoral response, and IFN-γ/IL-2 T-cell release in vitro than the individual treatment. rV-*neu*T+CUR-treated mice showed an increased infiltration of CD4^+^/CD8^+^ T lymphocytes within the tumor as compared to those that received the individual treatment. Overall, CUR enhanced the antitumoral effect and immune response to Neu induced by the rV-*neu*T vaccine in mice. Thus, the combined treatment might represent a successful strategy to target ErbB2/Neu-overexpressing tumors.

## 1. Introduction

The rate of head and neck cancer (HNC) is increasing worldwide, and despite improvements in treatment, the survival rate of HNC patients has not substantially changed in the last two decades [1]. The progress of novel therapeutic protocols can complement existing treatments for HNC patients [2]. Members of the epidermal growth factor receptor family (EGFR-ErbB4) have been involved in the development of human neoplasia [3]. The ErbB2 protein is overexpressed in several tumors [4,5,6,7,8]. Recombinant poxviruses expressing tumor antigens have been safely employed to vaccinate cancer patients [9,10,11,12,13,14,15,16,17]. We previously demonstrated that the intratumoral vaccination with a recombinant vaccinia virus encoding for ErbB2/Neu (rV-*neu*T) induced a strong antitumor response and antitumoral activity in mammary and salivary gland tumors overexpressing ErbB2/Neu in BALB-*neu*T mice [2,18]. The strongest antitumoral effect was achieved after two vaccinations and a dose of 10^8^ pfu in BALB-*neu*T mice with transplanted salivary gland tumors cells overexpressing ErbB2/Neu [2]. However, human clinical trials have revealed that repeated administrations of the poxviral vaccine increase neutralizing antibodies that prevent immune response against the recombinant antigen expressed by the virus genome [19]. Therefore, other vaccines or drugs may be required to boost the anti-Neu immune response induced by the rV-*neu*T vaccination [20,21,22,23,24]. One such option would be the use of an immunomodulatory polyphenol as a boost to hosts given one administration of rV-*neu*T.

Polyphenols (resveratrol (RES), apigenin (API), and curcumin (CUR)), have reproducibly shown antineoplastic activity via targeting ErbB2/Neu and other oncogenic pathways [25,26,27,28].

CUR (l,7-bis-(4-hydroxy-3-methoxyphenyl)-l,6-heptadiene-3,5-dione) is a non-flavonoid polyphenol purified from the rhizome of the plant *Curcuma longa*. CUR is a pleiotropic molecule that targets a variety of signal transduction pathways, having antitumor, anti-inflammatory, antioxidant, immunomodulatory, and antimicrobial activities in both rodents and humans [25,28,29,30,31,32,33,34]. Recently, different studies have demonstrated that CUR is able to modify the adaptive immune response in mice and humans, thus affecting the tumor microenvironment and production of cytokines [35,36,37,38,39,40,41]. CUR treatment resulted in the inhibition of PD-L1 and p-STAT3Y705 expression both in vitro and in vivo in tongue squamous cell carcinoma [42]. Additionally, the immunosuppressive tumor microenvironment was modified after CUR treatment [42]. Indeed, CUR encapsulated in liposomes, together with epicatechin gallate and RES (TriCurin), induced the repolarization of the milieu of HPV^+^ tumor-associated macrophages from an M2 state to an M1 phenotype and induced the intratumor recruitment of activated natural killer (NK) cells and cytotoxic T cells (CTL) in tumor-bearing mice [43]. CUR was shown to improve the therapeutic efficacy of Listeria-Mage-b vaccination in a breast cancer model and to inhibit the suppressive activity of regulatory T (Treg) cells and enhance the ability of T cells to kill cancer cells in tumor-bearing hosts [44]. Bisdemethoxycurcumin significantly increased intratumoral CD8^+^ T-cell infiltration, elevated the level of interferon (IFN)-γ in the blood, and decreased the number of intratumoral myeloid-derived suppressor cells (MDSC) in C56BL/6 mouse models bearing subcutaneous or lung-metastasized MB79 bladder cancer [45]. Lu et al. observed a highly significant inhibition of tumor growth matched with a strong CTL response and high amounts of IFN-γ. There was also a net decrease in the frequency of MDSC when an intracellular-labile amphiphilic CUR-based micelle delivery system (CUR-PEG) was administered in combination with a Trp2-based vaccine to treat B16F10 advanced melanoma in C57BL/6 mice [46]. An increase in CD8^+^ T cell and a decrease in Foxp3^+^ Treg cells were detected in the peritumoral area of HER2/neu^+^ TUBO-transplanted immunocompetent BALB/c mice treated with CUR [29]. After CUR treatment in LLC-tumor bearing mice, there was a delay in tumor growth and prolonged survival attributed to T-cell contributions. Indeed, low-dose CUR increased the frequency of CD4^+^ and CD8^+^ T lymphocytes in the spleens of immunocompetent tumor-bearing mice [47]. Treatment with CUR and API, in addition to inhibiting tumor growth of melanoma cells xenografted onto C57BL/6 mice, was able to inhibit IFN-γ-induced Programmed death-ligand (PD-L)1 expression and to enhance T-cell-mediated melanoma cell killing [48]. Two different studies demonstrated that CUR converted CD4^+^CD25^+^Foxp3^+^ Treg cells into IFN-γ-producing Th1 cells in lung and colon cancer patients [49,50].

Thus, to potentiate the effect of the vaccine and to reduce the number and doses of rV-*neu*T immunizations, the aim of this study was to determine whether CUR could improve the antitumor activity of the rV-*neu*T vaccine in BALB-*neu*T mice bearing salivary gland tumors. The rationale of using rV-*neu*T vaccine plus the signaling-pathway inhibitor CUR was based on the potential ability of CUR to increase the antitumor immune response induced by the rV-*neu*T vaccine and on the ability of CUR to have a potential direct additive anticancer effect when mixed together with the rV-*neu*T vaccine by targeting different signaling pathways. Indeed, CUR, similarly to anti-ErbB2/Neu antibodies, was able to inhibit ErbB2/Neu phosphorylation in cancer cells, thus causing apoptosis and inhibition of cancer cell growth [51]. Overall, we demonstrated that CUR enhanced the antitumoral effect and immune response to Neu induced by the rV-*neu*T vaccine in mice.

## 2. Materials and Methods

### 2.1. Reagents, Peptides, Cells, and Antibodies

Dimethyl sulfoxide (DMSO), CUR from *Curcuma longa*, sulforhodamine B (SRB), and concanavalin A (ConA) were purchased from Sigma-Aldrich (St. Louis, MO, USA).

Synthetic peptides located in the extracellular (Neu 15.3, aa 66–74-TYVPANASL; Neu 42, aa 169–183-DMVLWKDVFRKNNQL; Neu 98, aa 393–407-IAPLRPEQLQVFETL; Neu 141, aa 566–580-LPCHPECQPQNSSET; Neu 156, aa 626–640-GICQPCPINCTHSCV) or transmembrane (Neu 166, aa 666–680-VLLFLILVVVVGILI) domains of rat Neu sequence [52] were previously described [53].

NIH3T3 cells encoding normal rat Neu (LTR-Neu) were previously characterized and kindly provided by Dr. Eddi Di Marco (Istituto Tumori di Genova) [54]. Cell lines derived from HNCs of the tongue (SCC-15), pharynx (FaDu), or salivary gland (A-253) were maintained in RPMI containing 10% fetal bovine serum, 100 U/mL penicillin, and 100 μg/mL streptomycin. Neu-overexpressing salivary gland cancer cells (H-2d) (SALTO-5), established from a salivary carcinoma arising in BALB-*neu*T transgenic male mice, were kindly provided by Prof. F. Cavallo (University of Torino) and Prof. PL. Lollini (University of Bologna) and maintained in Dulbecco’s Modified Eagle Medium (DMEM) containing 20% fetal bovine serum (FBS) [55].

The purified mouse IgG1a (kappa) MOPC-21 was purchased from Cappel/Organon Teknika Corporation (West Chester, PA, USA) and used as a control. The mouse hybridoma cell line (Mouse Hybridoma, B109 4D5, PTA1624) producing the monoclonal antibody 4D5 was purchased by ATCC (Manassas, 20108 VA USA). Monoclonal antibody (mAb) 4D5 was then purified by protein G (Sigma-Aldrich, St. Louis, MO, USA).

### 2.2. Poxviruses

The recombinant vaccinia virus encoding the *neu* oncogene was designated rV-*neu*T (vT67RR-1-1, original lot from Therion Biologics Corp: #SC012197). It encodes the full length of the activated rat *neu* oncogene (NCI, PubMed Accession 1202344A) [52]. The wild-type control vaccinia virus was designated V-wt (original lot from Therion Biologics Corp: #062797-NYCBH). Therion Biologics Corp. (Cambridge, MA, USA) kindly provided the poxviruses [18]. rV-*neu*T was previously characterized [2].

### 2.3. Transgenic BALB-*neu*T Mouse Colony

Transgenic BALB-*neu*T male mice were routinely mated with BALB/c females (H-2d; Charles River, Calco, Italy) in the animal facilities of Tor Vergata. Progeny were confirmed for presence of the transgene by PCR [56]. Mice were bred under pathogen-free conditions and handled in compliance with European Union and institutional standards for animal research under protocols approved by the Italian Ministry of Health (authorization no. 844_2018-PR).

### 2.4. Recombinant Vaccinia neu Vaccination and Analysis of Antitumor Activity In Vivo

The investigation was conducted in accordance with the ethical standards and according to the Declaration of Helsinki. The work was conducted with the formal approval of the local [“Organismo Preposto al Benessere degli animali” (O.P.B.A.), University of Rome Tor Vergata] and national (Ministry of Health) animal care committees and animal experiments were registered as legislation requires (Authorization from the Ministry of Health no. 844_2018-PR issued on 23 October 2018). To determine the in vivo effect of the rV-*neu*T vaccination and CUR on the growth of SALTO-5 cells, groups of 6 to 8 weeks old BALB-*neu*T mice were subcutaneously inoculated in the right flank with 0.2 mL Phosphate-buffered saline (PBS) containing 1 × 10^6^ SALTO-5 cells. Intratumoral vaccination using 10^7^ pfu of rV-*neu*T or V-wt viruses in 100 µL of PBS (1×), was done when mice presented palpable masses one week after SALTO-5 transplantation. The rV-*neu*T vaccination was employed alone or in combination with the oral administration of CUR (2 mg/mouse in 50 µL corn oil, twice a week) or corn oil used as vehicle (50 µL, twice a week). Mice were inspected weekly for tumor growth. Tumors were measured in two directions (perpendicular diameters) using calipers and tumor volume (mm^3^) was calculated using the formula: (smaller diameter)^2^/larger diameter. When tumors exceeded an average diameter of 20 mm, the mice were sacrificed. Depending on the immunogen, groups of 3–8 mice were vaccinated: V-wt, n = 4; CUR, n = 3; corn oil, n = 4; V-wt+corn oil, n = 4; V-wt+CUR, n = 6; rV-*neu*T, n = 7; rV-*neu*T+corn oil, n = 8; rV-*neu*T+CUR, n = 8.

### 2.5. Antibody Immunity Following Vaccination with rV-*neu*T

The magnitude of the immune response elicited by the vaccination was evaluated quantitatively by ELISA (enzyme-linked immunosorbent assay). Mouse pre-immune or immune sera were collected prior to (T0) or three weeks after (T1) vaccination in tumor-bearing mice. Pooled mouse sera (two pools of three or four mice per each group) were analyzed at different dilutions (1:100, 1:500, 1:2500, 1:5000). To remove non-specific binding, sera were first incubated with NIH3T3 control cells (5 × 10^4^ cells/well) for two hours at 37 °C. Sera were then incubated with LTR-Neu (5 × 10^4^ cells/well) for 2 h at 37 °C. Antibody titer was estimated as the highest immune serum dilution generating a specific absorbance of 1.0 at 492 nm when reacted with LTR-Neu. No reactivity was observed with T0 sera.

Immunoglobulin subclasses were determined by ELISA using a Mouse Typer Isotyping Kit (Biorad, Richmond, CA, USA) using pooled sera of vaccinated mice (two pools of three mice per each group) as previously described [18]. To remove non-specific binding, sera were first incubated with NIH3T3 as described above.

To confirm the presence of antibodies reacting with Neu, sera were assayed by immunoprecipitation following immunoblotting using LTR-Neu cells as previously described [53]. Criteria of positivity comprised appearance of an immunoreactive band in the LTR-Neu transfectant comigrating with the one visualized by the anti-Neu polyclonal antibody on the LTR-Neu extract [53]. LTR-Neu cells were lysed in RIPA buffer containing 1% Triton-X100, 0.5% deoxicolate, 0.1 % SDS, 20 mM Tris pH 7.5, 150 mM sodium chloride, proteases, and inhibitors. Protein concentration was determined using the Bradford protein assay (Bio-Rad, Richmond, CA, USA), and 400 μg lysate of LTR-Neu transfectant was incubated with 2 μL of serum from mice in one of the treatment conditions and 50μL of protein G-sepharose. Immunoprecitates were separated on SDS-PAGE and transferred to nitrocellulose; the specific anti-Neu antibody was then added to the membrane (Santacruz Biotechnology, CA, USA) [27]. Lysate (80 μg) of LTR-Neu was used for Western blotting as a positive control. Two different pools of serum were used for immunoprecipitation. The intensities of the bands obtained were quantified using ImageJ software after blot scanning.

### 2.6. IL-2 and IFN-γ Release Assay

Spleen cells from BALB-*neu*T-vaccinated mice were harvested at sacrifice as previously described [53]. Spleen mononuclear cells (2 × 10^6^/well in 24 well plates) were incubated with concanavalin A (ConA, 2 μg/mL), an unrelated control peptide gag, or with various Neu peptides (10 μg/mL). Neu peptides were previously described [18,53,57]. Synthetic Neu peptides were selected based upon known immunogenicity in vitro for lymphocytes of BALB-*neu*T mice vaccinated with recombinant adenovirus expressing Neu or human ErbB2 [57]. Interleukin (IL)-2 and IFN-γ release into the supernatant were measured using an enzymatic immunocapture assay (Quantikine^®^, R&D Systems, Minneapolis, MN, USA). A few mice were randomly chosen from the different groups: CUR, n = 3; V-wt+CUR, n = 3; rV-*neu*T, n = 3; V-wt, n = 4; rV-*neu*T+oil, n = 4; rV-*neu*T+CUR, n = 4. Results represent three independent experiments of T-cell stimulation with peptides. Response to the gag unrelated peptide was subtracted from the response to the Neu peptides.

### 2.7. Histological Examination and Immunohistochemistry

Tumors from three animals from each group of mice were used for histological examination after hematoylin/eosin staining using 3 μm thick paraffin sections.

Immunohistochemistry (IHC) was used to analyze the leukocyte infiltration into tumors collected from three mice for each group. For IHC, antigen retrieval was performed on 3 μm thick paraffin sections using EDTA citrate, pH 7.8, or citrate pH 6.0 buffers for 30 min at 95 °C. Sections were then incubated for 1 h at room temperature with primary antibodies [anti-CD4: clone GK1.5, 1:100, (Thermo Fisher Scientific, Waltham, MA, USA); anti-CD8: clone YTS169.4, 1:100, (Thermo Fisher Scientific, Waltham, MA, USA); anti-CD19: clone SP110, 1:100, (R&D Systems, Minneapolis, MN, USA); anti-F4/80: clone CI:A3-1, 1:100, (BioXcell, Lebanon, NH, USA); polyclonal anti-cleaved caspase-3, 1:300, (Cell Signaling Technology, Leiden, NL), catalog #9661; anti-Gr-1/Ly-6G: clone RB6-8C5, 1:100, (Novus Biologicals, Centennial, CO, USA)]. To remove non-specific binding, slides were washed using PBS/Tween20, pH 7.6. Antibody–antigen binding was revealed by the Horseradish Peroxidase-3,3-diaminobenzidine (HRP-DAB) Detection Kit (UCS Diagnostic, Rome, Italy).

A digital scan was used to evaluate the immunohistochemical reactivity (Iscan Coreo, Ventana, Tucson, AZ, USA). A semi-automatic method based on the digital software Virtuoso (Ventana, Tucson, AZ, USA) was employed. By using this software, CD8, CD4, CD19, F4/80, Gr-1, and cleaved caspase-3 cells were counted by digitally labeling the number of positive cells in 10 high-power fields (20× or 40×) [58]; total cross-sectional area 5.5 mm^2^.

### 2.8. ErbB2 Expression in Human Cell Lines

MAb 4D5 was used to detect the level of ErbB2 expression in human HNC cell lines with flow cytometric analysis using a FACS Calibur cytometer. SCC-15, FaDu, and A-253 cell tumor cells (5 × 10^5^/tube) were labeled with the primary antibody anti-human ErbB2 mAb 4D5 (5 µg/mL) and then with FITC-labeled goat anti-mouse IgG (1:100) (catalog #115-095-146, Jackson Immunoresearch, Ely, UK). MOPC-21 (5 μg/mL) was used as control. Antibody binding was analyzed using Flowing Software 2.5.1.

### 2.9. Sulforhodamine B (SRB) Assay

For the cell proliferation assay, SCC-15, FaDu, and A-253 cells (7 × 10^3^ cells/well) were incubated for 48 and 72 h in serum-free RPMI containing 0.2% Bovine serum albumin (BSA) and mAb 4D5 (1.25, 2.5, or 5 µg/mL). MOPC-21 (5 μg/mL) was used as a control. The assay was then performed as previously described [59]. The percentage survival of the cultures treated with the mAb 4D5 was calculated by normalization of their Optical density (O.D.) values to those of control cultures. The experiments were performed in triplicate and repeated three times.

### 2.10. Statistical Methods

Continuous variables were summarized by mean and standard deviation, categorical variables were described by absolute frequencies and percentages. Continuous variables were compared by *t*-test.

Survival curves were estimated using the Kaplan–Maier method and compared using the Wilcoxon test. Multivariate analysis of survival times was based on the Cox model. Unknown parameters were estimated by maximum partial likelihood. Effects were tested using the Wald (chi-square) statistic.

In order to analyze the combined effect of CUR and rV-*neu*T vaccination on cancer volume over time, a mixed linear model was fitted to observed data. In our study, repeated measurements were taken on the same experimental unit, and these repeated measurements were correlated and exhibited variability that changed. To take these features into account, we introduced into the classical linear model a variance covariance matrix with a completely unspecified structure. The method of residual maximum likelihood was used to estimate unknown parameters. The significance of estimated effects was tested using the *t*-statistic.

Cytokine release, IHC data, and distribution of cell survival were preliminarily verified using the Kolmogorov–Smirnov test and data sets were analyzed by one-way analysis of variance (ANOVA) followed by Tukey’s test with GraphPad Prism 5.00.288. Values of *p* ≤ 0.05 were considered significant.

Differences in titers of the sera and isotypes of immunoglobulins were evaluated by a two-tailed Student’s *t*-test. Statistical associations were considered significant at *p*-values ≤ 0.05.

## 3. Results

### 3.1. CUR Potentiated the Effect of the rV-*neu*T Vaccination in Inhibiting the In Vivo Growth of SALTO-5 Cells Transplanted in BALB-*neu*T Mice

**Figure 1:**
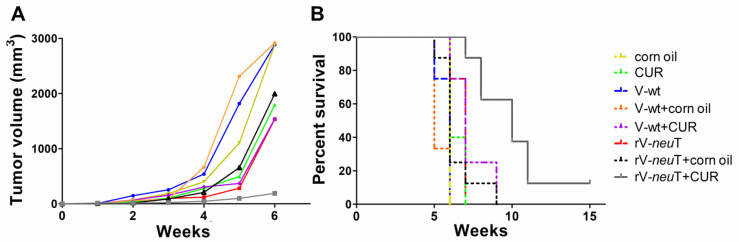
Growth inhibition of transplanted salivary gland (SALTO-5) tumor cells. (**A**) Differences in the mean tumor volume between BALB-*neu*T mice receiving corn oil or Curcumin (CUR) alone or in combination with V-wt or rV-*neu*T vaccinations. (**B**) Differences in the mean survival time of BALB-*neu*T mice receiving corn oil or CUR alone or in combination with V-wt or rV-*neu*T. Depending on the immunogen, groups of 3–8 mice were vaccinated: V-wt, n = 4; CUR, n = 3; corn oil, n = 4; V-wt+corn oil, n = 4; V-wt+CUR, n = 6; rV-*neu*T, n = 7; rV-*neu*T+corn oil, n = 8; rV-*neu*T+CUR, n = 8.

The simultaneous administration of rV-*neu*T+CUR was significantly superior in reducing tumor growth in vivo, compared to rV-*neu*T, rV-*neu*T+corn oil or CUR treatments. Indeed, the average tumor volume was 189.7 mm^3^ in rV-*neu*T+CUR treated mice versus 1533.3 mm^3^ in rV-*neu*T-treated mice (*p* = 0.0108) and versus 1991.2 mm^3^ in rV-*neu*T+corn oil-treated mice (*p* = 0.0069) 6 weeks after tumor challenge. Additionally, mice receiving rV-*neu*T+CUR showed reduced tumor volume in comparison to mice receiving CUR alone (1794.1 mm^3^, *p* = 0.0067) (Figure 1A).

To further investigate the combined effect of rV-*neu*T vaccination and CUR administration on tumor volume over time, a multivariable mixed linear model was fitted to our observations. No significant effect of corn oil administration on tumor growth was observed in any week (*p* = 0.0690). Therefore, in order to reduce estimated standard error, corn oil effect was not considered in the final multivariable model. Table 1 shows the results of this multivariable mixed linear model on tumor volume over time. In the first part of Table 1, this estimated tumor growth over time is shown for the V-wt control group. Expected tumor volume started to increase at Week 2, and growth rate sharply rose from Week 4 to Week 6; expected volume at Week 6 was about 3019 mm^3^ (Table 1).

A significant reduction of the tumor volume was observed in rV-*neu*T- as compared to V-wt-vaccinated mice; this reduction was already significant 2 weeks after vaccination (*p* = 0.0001) and increased over time (Table 1), reaching a decrease of −1205.78 mm^3^ at 6 weeks, when the expected volume in rV-*neu*T group was about 1813 mm^3^.

The CUR antitumoral effect became significant 4 weeks after the beginning of the treatment and increased over time. At 6 weeks, the estimated tumor reduction due to CUR administration, as compared to V-wt-vaccinated mice, was −1576.84 mm^3^ and the expected tumor volume in the CUR group was about 1443 mm^3^.

It is worth of noting that, when considering the simultaneous rV-*neu*T+CUR treatment, the antitumoral effect of the CUR treatment was not dependent on that exerted by the rV-*neu*T vaccination, resulting in no significant interaction between the two treatments (*p* = 0.7047). The reduction in volume due to the CUR treatment added to the estimated reduction due to the rV-*neu*T vaccination, leading to an expected reduction of volume for rV-*neu*T+CUR versus V-wt at 6 weeks of −2782.62 mm^3^ (*p* < 0.0001). The estimated tumor volume in the rV-*neu*T+CUR group at 6 weeks was about 237 mm^3^.

Upon univariate descriptive analysis, the survival probability was significantly higher in mice vaccinated with rV-*neu*T+CUR than in those receiving rV-*neu*T (median survival time of 10 versus 7 weeks *p* = 0.0036), rV-*neu*T+corn oil (median survival time of 10 versus 6 weeks *p* = 0.0012), and CUR (median survival time of 10 versus 6 weeks *p* = 0.0012) (Figure 1B). Overall, when comparing the survival of BALB-*neu*T mice after treatments, the estimated hazard ratio (HR) was 27.45 for rV-*neu*T, 12.01 in rV-*neu*T+corn oil and 28.90 in CUR in comparison to rV-*neu*T+CUR-treated mice.

Upon multivariate analysis, in order to analyze the antitumor effect of combined rV-*neu*T+CUR treatment, the Cox model was fitted to the observed data. The main effects of rV-*neu*T, CUR and corn oil were evaluated together with the potential interaction between rV-*neu*T and CUR. No significant effect of corn oil was observed (*p* = 0.4641). Estimated hazard ratios (HR) were 6.45 (*p* = 0.0017) and 11.85 (*p* < 0.0001) for V-wt vs. rV-*neu*T and rV-*neu*T vs. rV-*neu*T+CUR, respectively (Table 2). The interaction between CUR and rV-*neu*T was not significant (*p* = 0.5846), thus corroborating the multivariable mixed linear model analysis and indicating that the antitumoral effect of CUR was additive to that of rV-*neu*T. The estimated HR for V-wt vs. rV-*neu*T+CUR was 76.377 (*p* < 0.0001) (Table 2).

Overall, these results indicate that CUR administration potentiated the antitumoral effect of the rV-*neu*T vaccination.

### 3.2. CUR Increased the Anti-Neu Humoral Response Induced by the rV-*neu*T Vaccination

In order to evaluate whether CUR induced an increase in the anti-Neu humoral immune response elicited by the rV-*neu*T vaccination, the specific anti-Neu antibody response was evaluated by ELISA. rV-*neu*T and rV-*neu*T+corn oil immunizations were effective in inducing a high antibody titer to ErbB2/Neu as compared to V-wt vaccination. However, when rV-*neu*T vaccination was combined with the CUR administration, the titer of antibodies to Erb2/Neu was increased up to 1:5000 as compared to rV-*neu*T (1:2100, *p* < 0.0001) and rV*neu*T+corn oil (1:2400, *p* < 0.0001) (Table 3). Therefore, CUR increased the anti-Neu humoral response induced by the rV-*neu*T vaccination. The administration of corn oil, CUR, and V-wt, alone or combined with CUR did not result in the induction of anti-Neu antibodies.

**Figure 2:**
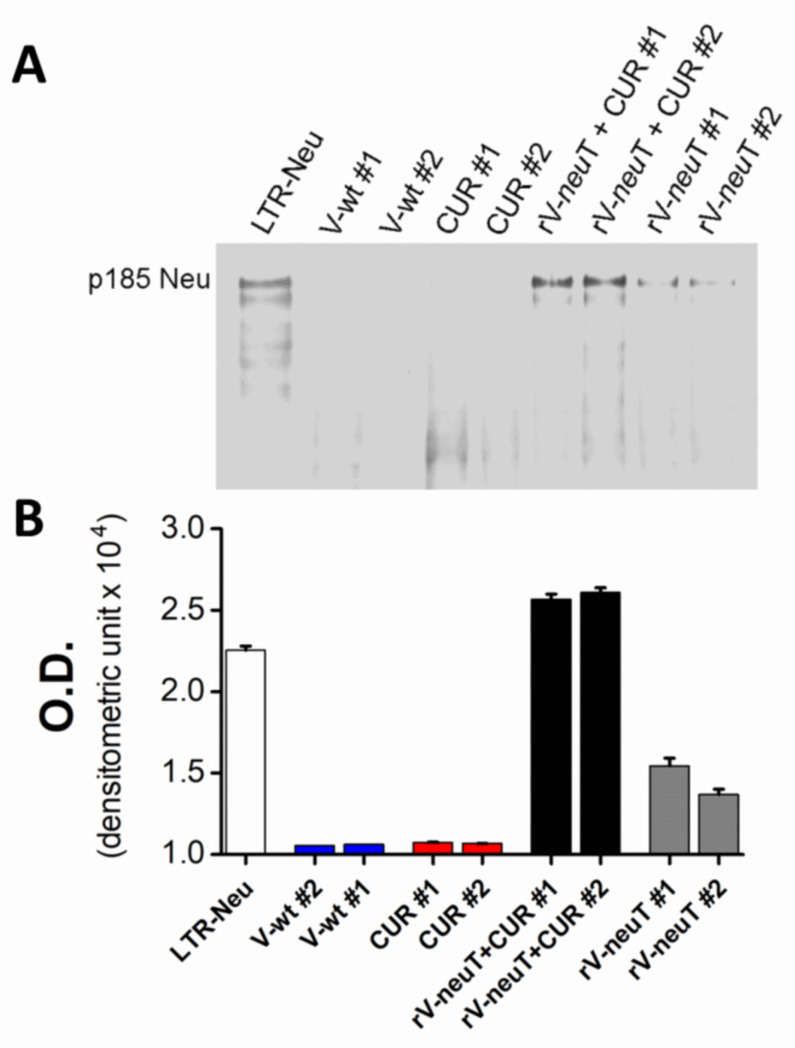
Serum antibody response of mice upon treatments with V-wt, Curcumin (CUR), rV-*neu*T, or rV-*neu*T+CUR. (**A**) Induction of serum antibodies specific for Neu following treatments was detected by immunoprecipitation following Western blotting. Sera from three to four mice for each group were pooled (Group #1, Group #2) and used to immunoprecipitate LTR-Neu cell lysate. Neu specificity was visualized by Western blotting analysis using a specific anti-Neu polyclonal antibody (80 μg LTR-Neu cell lysate was loaded for Western blotting analysis). (**B**) The intensities of the bands obtained were quantified using ImageJ software after blot scanning.

The presence of specific antibody to Neu in the serum of rV-*neu*T-vaccinated mice was qualitatively investigated by immunoprecipitation following Western blotting using sera from mice vaccinated with V-wt, CUR, rV-*neu*T, or rV-*neu*T+CUR respectively (Figure 2). Sera from three to four mice for each group were pooled (Group #1 and Group #2) and used to immunoprecipitate the cell lysate from LTR-Neu transfectants. Specific reactivity was visualized by Western blotting of immunoprecipitates using the anti-Neu specific polyclonal antibody. Specific antibodies against Neu were detected in mice vaccinated with rV-*neu*T, and rV-*neu*T+CUR. No reactivity was detected in mice vaccinated with V-wt or treated with CUR (Figure 2A). Mice vaccinated with rV-*neu*T+CUR showed a stronger reactivity against Neu compared to mice immunized with rV-*neu*T alone (Figure 2B).

There was no pronounced shift in distribution of Neu-ECD specific immunoglobulin subclasses after the rV-*neu*T+CUR treatment as compared to treatment with rV-*neu*T or rV-*neu*T+corn oil (Table 4).

### 3.3. CUR Increased T-Cell Immune Response Induced by rV-*neu*T Vaccination

In order to determine whether the simultaneous administration of CUR modified T-cell response against Neu induced by the rV-*neu*T vaccination, splenocytes isolated from mice treated with different protocols were examined for their ability to proliferate in the presence of various Neu peptides. The release of IL-2 and IFN-γ was measured in the supernatant to assess T-cell immunoreactivity with specific Neu epitopes. The values of cytokines released by T cells upon the unrelated negative control peptide gag stimulation were subtracted from those obtained upon the Neu peptide stimulation. ConA was used as positive control, and T-cell proliferative response upon ConA stimulation was similar for all the vaccinated groups.

**Figure 3:**
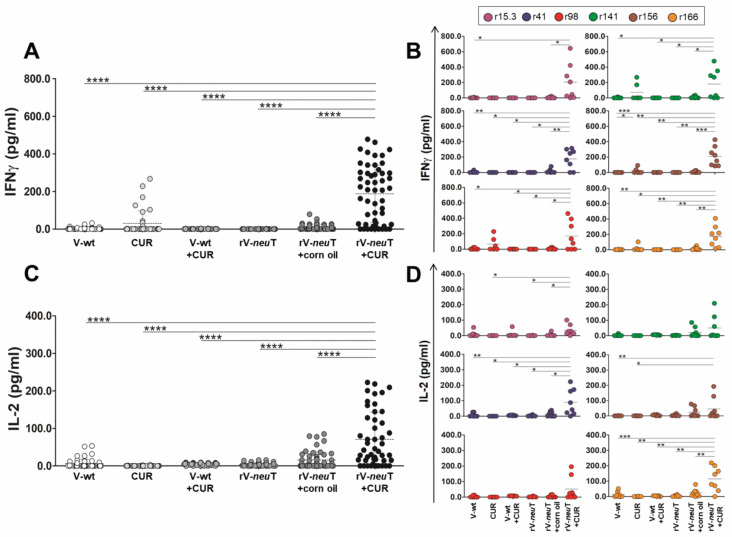
Interferon (IFN)-γ and Interleukin (IL)-2 release from splenocytes treated with Neu peptides. The release of cytokines was assessed in splenocytes collected at sacrifice from treated mice after 96 h stimulation in vitro with Neu peptides. The results show the cytokine release induced by an irrelevant peptide (gag) subtracted from the IFN-γ or IL-2 release of each sample, in two experiments performed in duplicate (*p ≤ 0.05, ** *p* ≤ 0.01, *** *p* ≤ 0.001, **** *p* ≤ 0.0001; one-way-ANOVA, Tukey’s multiple comparison). (**A**,**C**) show the release of IFN-γ or IL-2 when all peptides were used in the assay, while (**B**,**D**) show the contribution of each peptide to the T-cell cytokine release. Results represent three independent experiments of T-cell stimulation with Neu peptides.

Neu peptides stimulation induced higher IFN-γ and IL-2 release by T cells from rV-*neu*T+CUR-treated mice than in those from rV-*neu*T- or CUR-treated mice (Figure 3A,C). The IFN-γ release by T cells from rV-*neu*T+CUR-treated mice was significantly higher than the release by T cells from rV-*neu*T-, rV-*neu*T+corn oil-, or CUR-treated mice when stimulated with all peptides, and particularly with the r156 peptide (Figure 3B). Conversely, IL-2 released by T cells from mice treated with rV-*neu*T+CUR was significantly higher than the release by T cells from rV-*neu*T-, rV-*neu*T+corn oil-, or CUR-treated mice when stimulated only with the r41 and r166 peptides (Figure 3D). Very low or undetectable cytokine release was obtained after Neu peptide stimulation by T cells from mice immunized with CUR, V-wt, or the combination of these two.

### 3.4. CUR Increased Necrotic Areas and Inflammatory Cell Infiltration into SALTO-5 Tumors of rV-*neu*T-Vaccinated Mice

**Figure 4:**
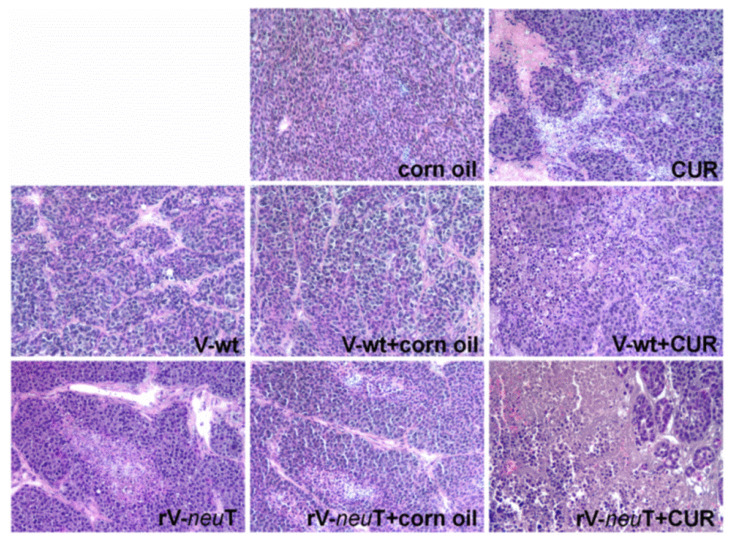
Histology of tumors from mice treated with V-wt, Curcumin (CUR), rV-*neu*T, or the combination. Tumors from three different animals for each group were stained using hematoxylin and eosin. Original magnification 100×.

Tumors from mice treated with corn oil, CUR, V-wt, V-wt+corn oil, V-wt+CUR, rV-*neu*T, rV-*neu*T+corn oil, or rV-*neu*T+CUR were processed for paraffin embedding. Tumors arising from three different animals for each group were examined. Histological examination of tumors revealed the presence of small areas of necrosis in mice treated with CUR or V-wt+CUR. Focal areas of necrosis were evident in mice vaccinated with rV-*neu*T. Tumors from rV-*neu*T+CUR-treated mice showed remarkable necrosis characterized by abundant cellular debris as compared to rV-*neu*T- and CUR-treated mice. Necrosis was not detected in mice vaccinated with V-wt, V-wt+corn oil, or treated with corn oil (Figure 4).

**Figure 5:**
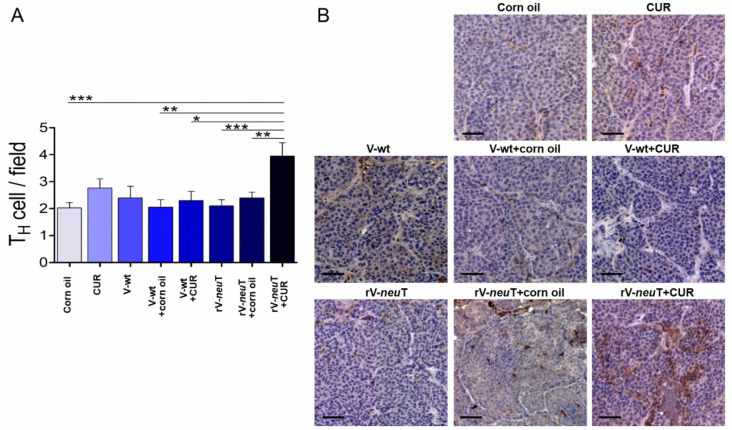
Helper T cells infiltrating tumors in BALB-*neu*T mice after treatments. Tumor tissues from three mice in each group were analyzed with IHC for CD4 expression. (**A**) Positive cell count/field averaging 10 representative microscopic fields (mean ± SD, * *p* ≤ 0.05, ** *p* ≤ 0.01, *** *p* ≤ 0.001; one-way-ANOVA, Tukey’s multiple comparison). (**B**) Representative digital images (20×), scale bar represents 100 µm. T_H_: helper T lymphocytes, CUR: curcumin.

**Figure 6:**
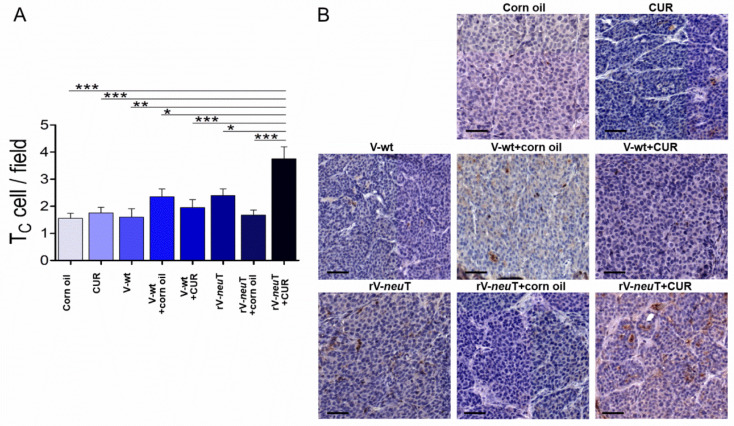
Cytotoxic T cells infiltrating tumors in BALB-*neu*T mice after treatments. Tumor tissues from three mice in each group were analyzed with IHC for CD8 expression. (**A**) Positive cell count/field averaging 10 representative microscopic fields (mean ± SD, * *p* ≤ 0.05, ** *p* ≤ 0.01, *** *p* ≤ 0.001; one-way-ANOVA, Tukey’s multiple comparison). (**B**) Representative digital images (20×), scale bar represents 100 µm. T_C_: cytotoxic T lymphocytes, CUR: curcumin.

Immunohistochemical (IHC) analysis was performed to detect the presence of tumor-infiltrating leukocytes (TILs) in tumors from BALB-*neu*T-treated mice. Samples were evaluated on digital images by counting the number of positive cells in 10 high-power fields (HPFs) (40×). Necrotic areas were excluded from the analysis. Tumor tissue analysis showed an important infiltration of T-helper lymphocytes (T_H_, CD4^+^) (Figure 5). Post-hoc analysis (Tukey’s test) showed a significant increase in the T_H_ cells within the tumor in rV-*neu*T+CUR-treated (4.0 ± 2.2) mice as compared to rV-*neu*T+corn oil- (2.4 ± 1.5; *p* < 0.01), rV-*neu*T- (2.1 ± 1.3; *p* < 0.001), V-wt+CUR- (2.3 ± 1.5; *p* < 0.05), V-wt+corn oil- (2.1 ± 1.3; *p* < 0.01), and corn oil-treated (2.0 ± 1.3; *p* < 0.001) mice (Figure 5A). T_H_ cells were homogeneously distributed in the tumors of rV-*neu*T+CUR treated mice (Figure 5B). A considerable infiltration of T-cytotoxic lymphocytes (T_C,_ CD8^+^) was observed in mice treated with rV-*neu*T+CUR (Figure 6). A post-hoc test showed a significant increase in the number of Tc cells within the tumor in rV-*neu*T+CUR-treated (3.8 ± 2.0) mice as compared to mice receiving other treatments: rV-*neu*T+corn oil (1.7 ± 1.2; *p* < 0.001), rV-*neu*T (2.4 ± 1.3; *p* < 0.05), V-wt+CUR (2.0 ± 1.3; *p* < 0.001), V-wt+corn oil (2.4 ± 1.3; *p* < 0.05), V-wt (1.6 ± 1.0; *p* < 0.01), CUR (1.8 ± 1.4; *p* < 0.001), or corn oil (1.6 ± 1.2; *p* < 0.001) (Figure 6A). Notably, clusters of T_C_ cells, rather than homogeneously dispersed cells, were observed within the tumors from rV-*neu*T+CUR-treated mice (Figure 6B). Tumors from all groups of treated mice showed the presence of both B-cell lineage (CD19^+^, Supplementary Figure S1) and macrophages (Mf, F4/80^+^, Supplementary Figure S2). However, no significant differences were detected among different groups. Neutrophil infiltration was observed in tumors from mice treated with rV-*neu*T+CUR (Supplementary Figure S3). There was not any difference between the number of tumor neutrophils in rV-*neu*T+CUR-treated (2.8 ± 0.8) mice as compared to that in CUR- (1.7 ± 1.1), rV-*neu*T- (1.9 ± 1.0) or rV-*neu*T+corn oil-treated (1.8 ± 0.6) mice (Supplementary Figure S3). 

**Figure 7:**
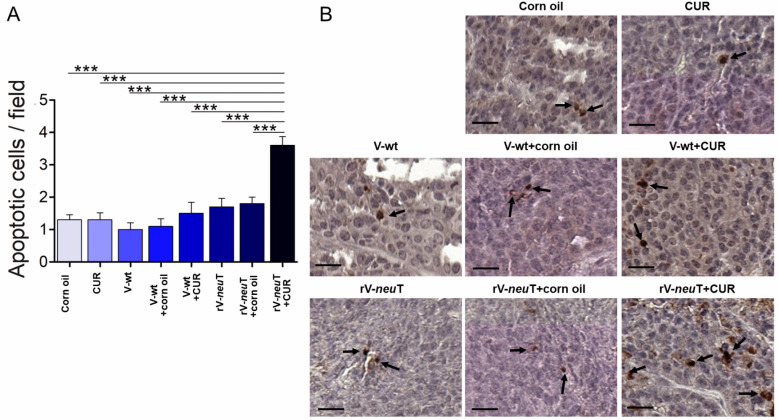
Apoptotic cells within the tumors of BALB-*neu*T-treated mice. Tumor tissues from three mice in each group were analyzed by IHC analysis for cleaved caspase-3 expression (arrow). (**A**) Positive cell count/field averaging 10 representative microscopic fields (mean ± SD, *** *p* ≤ 0.001; one-way-ANOVA, Tukey’s multiple comparison). (**B**) Representative digital images (40×), scale bar represents 50 µm. CUR: curcumin.

The presence of apoptotic cells was evaluated by cleaved caspase-3 IHC analysis (Figure 7). The number of apoptotic cells within the tumors from rV-*neu*T+CUR-treated (3.6 ± 0.8) mice was higher than that from rV-*neu*T+corn oil- (1.8 ± 0.6, *p* < 0.001), rV-*neu*T- (1.7 ± 0.8, *p* < 0.001), V-wt+CUR- (1.5 ± 1.1; *p* < 0.001), V-wt+corn oil- (1.1 ± 0.7; *p* < 0.001), V-wt- (1.0 ± 0.7; *p* < 0.001), CUR- (1.3 ± 0.7, *p* < 0.001), and corn oil-treated (1.3 ± 0.5; *p* < 0.001) mice.

### 3.5. Biological Effects of mAb 4D5 on HNC Cells

We previously demonstrated that immunoglobulins from mice vaccinated with rV-*neu*T were able to inhibit cell proliferation, to trigger cell apoptosis, and to decrease ERK1 phosphorylation in SALTO-5 cells [2]. Here, we demonstrated that the addition of CUR increased the titer of anti-Neu immunonglobulins in BALB-*neu*T mice and that the increased anti-Neu immunoglobulins concentration was concomitant with the decrease in the growth of SALTO-5 cells transplanted into BALB-*neu*T mice. In view of the in vivo and in vitro effects of the anti-Neu immunoglobulins on SALTO-5 cell growth, the role of the anti-ErbB2/Neu mAb 4D5 in modulating the growth of human HNC cells lines overexpressing ErbB2 was investigated.

**Figure 8:**
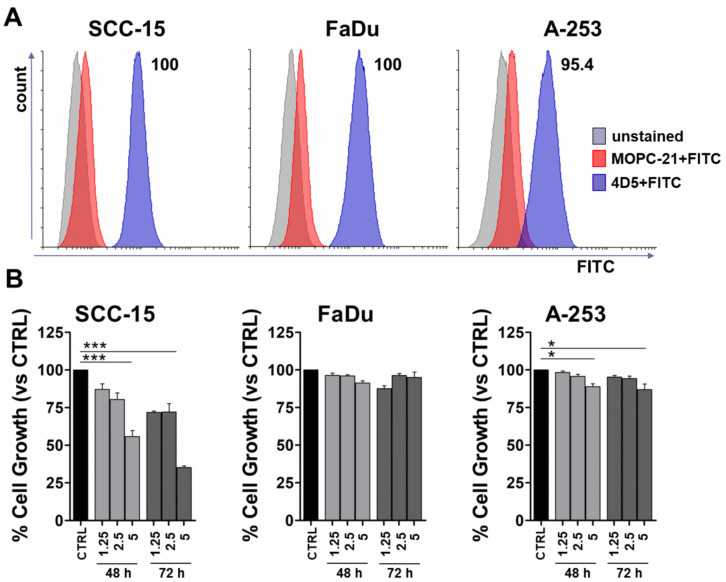
In vitro effect of mAb 4D5 on Head and neck cancer (HNC) cells. (**A**) FACS analysis was performed on HNC cell lines of the tongue (SCC-15), pharynx (FaDu), or salivary gland (A-253) using mAb 4D5 (5 μg/mL). MOPC-21 (5 μg/mL) was used as a control. (**B**) Cell growth was assessed by a sulforhodamine B (SRB) assay after 48 and 72 h of treatment with mAb 4D5 at different concentrations (1.25, 2.5, 5 μg/mL). MOPC-21 (5 μg/mL) was used as a control (CTRL). The results are expressed as the mean ± SD of three independent experiments performed in triplicate (* *p* ≤ 0.05, *** *p* ≤ 0.001, compared with the control cultures; one-way-ANOVA, Tukey’s multiple comparison).

The expression of ErbB2/Neu in different HNC cell lines (SCC-15, FaDu, A-253) was evaluated by FACS analysis employing mAb 4D5. MOPC-21 (5 μg/mL) was used as a control. All SCC-15 and FaDu cells were positive for mAb 4D5 binding, while 95.4% of A-253 cells were positive for ErbB2/Neu expression (Figure 8A). The mean fluorescence intensity (MFI) after mAb 4D5 binding was different between SCC-15 (MFI = 85.55), FaDu (MFI = 146.56) and A-253 (MFI = 53.38) cell lines.

In order to evaluate whether the treatment with mAb 4D5 was able to inhibit the growth of HNC cells, SCC-15, FaDu, and A-253 cells were seeded in serum-free culture medium containing 0.2% BSA and incubated with mAb 4D5 at different concentrations (1.25, 2.5, and 5 μg/mL) for 48 and 72 h. The antibody MOPC-21 (5 μg/mL) was used as a control.

MAb 4D5 inhibited cell growth in a dose- and time-dependent manner in SCC-15 cells compared to control (*p* < 0.001). Conversely, FaDu cell proliferation was not affected by mAb 4D5 treatment. A lower, but significant, decrease of A-253 cell proliferation was observed only at the highest concentration after 48 and 72 h of incubation (*p* < 0.05).

The means of the results of three independent experiments are reported in Figure 8B.

## 4. Discussion

The immunomodulatory properties of CUR have been reported in several studies. CUR qualitatively and quantitatively affects T cells, B cells, macrophages, neutrophils, NK cells, dendritic cell number, and the production of cytokines and chemokines in inflammatory and immune- mediated diseases [60]. The immunomodulatory properties of CUR were demonstrated in a two weeks treatment of 30 non-small-cell lung cancer patients. An increase of Th1 cells in peripheral blood and a decrease of Treg cells, as compared to untreated patients, was observed [49]. Similar results, indicating a possible conversion of Treg cells to Th1 cells due to CUR administration, were reported in a different study performed on 40 patients with colon cancer treated for 1 month with CUR after surgical removal of the tumor [50]. Some studies indicate that CUR exhibits immunosuppressive properties on lymphocytes [61,62]. The modulation of the immune response by CUR appears to be dependent on the dose and on the normal/abnormal behavior of the immune cells. Indeed, a low dose of CUR enhanced the proliferation of splenic lymphocytes, while a high dose of CUR decreased it [63]. Indeed, Luo et al. showed that a high dose of CUR decreased T cells, whereas a low dose treatment increased CD8^+^ T cells derived from 3LL tumor-bearing mice [47]. CUR promoted the proliferation of normal B cells in the mucosa of intestine of C57BL/6J-Min/+ (Min/+) mice [64]. On the other hand, CUR has been found to reduce the proliferation of immature B-cell lymphoma (BKS-2) cells [65]. In addition, CUR was shown to affect the proliferation and activation of cells involved in innate immunity, increasing the phagocytosis of macrophages [63] by differentially activating them through the downregulation of Th1 and nitric oxide (NO) production [66] and by increasing NK-cell cytotoxicity [61]. Overall CUR, by inactivating NF-κB, inhibits the expression of several proinflammatory cytokines [e.g., Tumor Necrosis Factor (TNF), IL-1, IL-2, IL-6, IL-8, and IL-12] and downregulates the mRNA of several pro-inflammatory enzymes [e.g., Cyclooxygenase (COX), Lipoxygenase (LOX), Matrix metalloproteinases (MMPs), and Nitric oxide synthase (NOS)] [67]. In a mouse model of colitis, CUR treatment decreased the expression of TNF-α, IL-2, IL-6, IL-12 p40, IL-17, and IL-21 to a level comparable to healthy mice [68]. CUR has been reported to be a direct binder and inhibitor of IL-2, preventing its binding to the IL-2 receptor α, CD25 [69], and interfering with IL-2 downstream signaling [JAK/STAT (Janus kinase/signal transducer and activator of transcription) pathway, NF-κB activation, and Foxp3 expression] [70,71]. It was recently demonstrated that daily administration of CUR significantly increased CXCR5^+^ B-cell lymphoma 6^+^ T_FH_ cells and CD95^+^ GL-7^+^ germinal center (GC) B cells in draining lymph nodes, and that CUR treatment in mice induced total antibody production as well as high-affinity IgG1 and IgG2b antibody production [72]. In rats fed with a CUR-supplemented diet, IgA secretion increased in the gut lumen, while serum IgA was decreased and serum levels of IgE and IgG were untouched [73].

In this study, we provided evidence that CUR potentiates the effect of the rV-*neu*T vaccination in inhibiting the in vivo growth of Neu-overexpressing salivary gland cancer cells (SALTO-5) transplanted in syngenic *neu*-tolerant BALB-*neu*T mice. The survival probability was significantly higher in mice treated with rV-*neu*T+CUR than in those receiving CUR, rV-*neu*T or rV-*neu*T+corn oil. Six weeks after tumor challenge, the average tumor volume in rV-*neu*T+CUR mice was 189.7 mm^3^ versus 1533.3 mm^3^ in rV-*neu*T mice, 1991.2 mm^3^ in rV-*neu*T+corn oil mice, and 1794.1 mm^3^ in CUR mice. Additionally, the survival probability in mice vaccinated with rV-*neu*T+CUR was 10 weeks, as compared to 7 weeks for mice vaccinated with rV-*neu*T and 6 weeks for those receiving CUR or rV-*neu*T+corn oil. Lastly, statistical models analyzing the increase of tumor volume and mouse survival unanimously indicated the additive antitumoral effect seen when CUR was administered together with the rV-*neu*T vaccine.

We previously demonstrated that rV-*neu*T vaccination generated a high immune response depending on the number of recombinant vaccinia virus vaccinations administered and on the amount of virus injected in mice [2,18].

A single injection of 10^7^ pfu of rV-*neu*T combined with prolonged administration of CUR was able to induce higher serum levels of anti-Neu antibodies (1:5000) as compared with that generated by rV-*neu*T alone (1:2100) or combined with vehicle (1:2400). Accordingly, the higher magnitude of anti-Neu humoral response induced by rV-*neu*T+CUR administration paralleled the stronger antitumor activity induced by the combined treatment, therefore contributing to the reduction of tumor growth. Several mechanisms have been identified as responsible for the inhibitory effect of anti-ErbB2/Neu antibodies in tumor cells expressing ErbB2/Neu: antibody-dependent cellular cytotoxicity (ADCC), complement-dependent cytotoxicity (CDC), and apoptosis induction or downregulation of receptor [74,75,76,77,78,79].

However, previous studies demonstrated that CUR not only enhanced B-cell function but also affected immunoglobulin isotype class-switching [72,80,81]. In our study, rV-*neu*T+CUR treatment was not able to affect immunoglobulin isotype class-switching, although it increased the titer of anti-Neu antibodies in mouse serum as compared to the single treatment. In addition, oral administration of CUR alone was not effective in raising anti-Neu antibody production. We previously demonstrated that Igs from rV-*neu*T mice were able to inhibit in vitro cell proliferation, inducing ADCC and apoptosis of SALTO-5 tumor cells [2]. In addition, it has been demonstrated that trastuzumab, the clinically used anti-ErbB2 mAb, is able to downregulate ErbB2 activity [82]. Therefore, we evaluated whether mAb 4D5 was able to inhibit cell proliferation of HNC cell lines of different origins. Our results showed that the anti-proliferative effect of mAb 4D5 was dependent on the type of cell line. Indeed, we found that an SCC-15 cancer cell line derived from the tongue was the most sensitive to the mAb treatment. Conversely, the FaDu cancer cell line derived from the pharynx was unaffected by mAb 4D5 treatment, and the A-253 cancer cell line, derived from salivary gland, showed a mild inhibition of the proliferation after incubation with mAb 4D5. The effect of mAb 4D5 was not dependent on ErbB/Neu expression, since all cells express ErbB2/Neu with the MFI ranging from 53 to 146, as we demonstrated by FACS analysis. In agreement with these results, it can be supposed that the effect of the humoral response to ErbB2/Neu, although potent, might not be efficient in counteracting the growth of certain types of cancer cells [83].

Consistent with previous evidence [2], we found that T cells purified from the spleens of rV-*neu*T-vaccinated mice released IFN-γ and IL-2 upon stimulation with Neu-specific peptides. After stimulation with all peptides, particularly the r156 peptide, the IFN-γ released by T cells from rV-*neu*T+CUR-treated mice was significantly higher than that released by T cells from mice that received treatment with a single compound. Conversely, after stimulation with peptides r41 and r166 only, IL-2 released by T cells from mice treated with rV-*neu*T+CUR was significantly higher than that released by T cells from mice in other group of treatment. Accordingly, the combined treatment potently stimulated the immune response to the r156 peptide. It worth noting that the peptide r156 is located in the extracellular domain of the rat sequence. IFN-γ has been shown to be involved in tumor rejection through deprivation of blood supply [84]. Recognition of Neu epitopes in vivo might potentially activate T cells to produce IFN-γ, thus causing ischemic necrosis at the tumor site.

Bhattacharyya et al. demonstrated that CUR has a role in reversing tumor-induced immune dysfunction, preventing the loss of T cells, restoring T cell proliferative and killing capacity, and expanding memory and effector T-cell populations. In addition, CUR switched the Th2-type to the Th1-type response by increasing IFN-γ secretion and downregulating the production of TGF-β and IL-10, thus inhibiting the suppressive activity of Treg [37]. Other in vivo studies reported recovery or increase in the levels of circulating or tumor-infiltrating CD4^+^ and CD8^+^ T cells and of NK cells, and a decreased number of tumor-infiltrating MDSCs after administration of CUR or a CUR-based cocktail [43,45,85]. In agreement with these studies, we demonstrated that the best antitumor activity was obtained using the rV-*neu*T+CUR combined treatment, which was reflected in significant changes of TILs. Indeed, CUR significantly increased the number of CD4^+^ and CD8^+^ T lymphocytes within the transplanted tumor when administered together with the rV-*neu*T vaccine. Synergy of B- and T-cell immunity is essential for eradication of Neu-expressing tumors [86]. The increase of CD8^+^ T lymphocytes induced by CUR in combination with the rV-*neu*T vaccine must be emphasized because, as we have reported above, the anti-Neu humoral response may not be effective for the elimination of cancer cells. TILs might be directly involved in tumor rejection, as demonstrated by the presence of apoptotic cells. In conjunction with anti-Neu antibodies, they might locally accelerate tumor cell elimination or release cytokines with antiangiogenetic properties, which mediate ischemic necrosis at the tumor site [87]. Larger areas of necrosis were observed in tumors from mice treated with the rV-*neu*T+CUR combined treatment.

Current therapeutic guidelines recommend trastuzumab application only in initial or metastatic breast cancer, metastatic gastric cancer, and gastroesophageal junction cancers when they overexpress the ErbB2 protein or with the amplification of the *ERBB2* gene (www.ema.europa.eu). A variety of tumors has been positively analyzed for ErbB2 overexpression or gene amplification: non-small-cell lung cancer, ovarian cancer, bladder cancer, pancreatic cancer, and salivary duct carcinoma [88]. Multiple lines of evidences showing efficacy of anti-HER2-targeted therapy have been reported for salivary duct carcinoma [89]. Given the experience with breast cancer, the monotherapy regimen is probably not preferable for salivary duct carcinomas or more generally for HNCs [88]. The combination of trastuzumab and chemotherapy was evaluated on a small sample of patients with salivary gland cancer, and there were encouraging results showing increased life expectancy, but the response was not long-lasting [90,91,92]. Active immunization targeting ErbB2 might sustain tumor inhibition more effectively than passive immunotherapy based on the stimulation of a sustained memory immune response. It would also be valid to boost a naturally occurring ErbB2 immune response. Moreover, active immunization using ErbB2 as immunogen might be advantageous over a single mAb monotherapy by simultaneously eliciting T- and B-cell immunity to multiple immunodominant epitopes.

Overall, we demonstrated that CUR enhanced the antitumoral effect and immune response to neu induced by the rV-*neu*T vaccine in mice. Thus, the combined treatment might represent a successful strategy to target ErbB2/Neu-overexpressing tumors.

## Figures and Tables

**Table 1:** Estimated effect on tumor volume over time according to the multivariable mixed linear model.

	**Time (Week)**	**Estimated Tumor Volume Over Time (mm^3^)**	**Standard Error**		
**V-wt**	**1**	7.54	2.69		
	**2**	103.11	14.72		
	**3**	191.62	32.08		
	**4**	556.47	57.88		
	**5**	1737.03	196.42		
	**6**	3019.57	295.22		
		**Expected Reduction of Tumor Volume (mm^3^)**	**Standard Error**	***t*-Statistic**	***p-*Value**
**CUR**	**1**	2.14	3.01	0.72	0.4793
	**2**	−19.56	16.46	−1.19	0.2435
	**3**	−54.09	35.87	−1.51	0.1417
	**4**	−186.18	64.71	−2.88	0.0072
	**5**	−857.72	219.60	−3.91	0.0005
	**6**	−1576.84	289.38	−5.45	<0.0001
**rV-*neu*T**	**1**	2.65	3.06	0.87	0.3930
	**2**	−75.01	16.75	−4.48	0.0001
	**3**	−106.11	36.52	−2.91	0.0067
	**4**	−356.26	65.87	−5.41	<0.0001
	**5**	−1035.72	223.56	−4.63	<0.0001
	**6**	−1205.78	312.21	−3.86	0.0005

**Table 2:** Estimated hazard ratios from the fitted Cox model.

			HR ^a^	95% CI ^b^	*p*-Value
**V-wt**	vs.	**rV-*neu*T**	6.447	(2.013, 20.642)	0.0017
**rV-*neu*T**	vs.	**rV-*neu*T+CUR**	11.848	(3.561, 39.414)	<0.0001
**V-wt**	vs.	**rV-*neu*T+CUR**	76.377	(9.690, 602.00)	<0.0001

^a^ HR: hazard ratio; ^b^ CI: confidence interval.

**Table 3:** Immunoreactivity of rV-*neu*T-vaccinated BALB-*neu*T mouse sera with Neu.

Group	Number of Pooled Sera	Serum Titer (SD) ^a^	*p*-Value
**Corn Oil**	4	Neg	
**CUR**	4	Neg	
**V-wt**	4	Neg	
**V-wt+Corn Oil**	3	Neg	
**V-wt+CUR**	4	Neg	
**rV-*neu*T**	6	2100 ^b^ (±0.012)	0.000001 ^c^
**rV-*neu*T+Corn Oil**	8	2400 (±0.020)	0.00001 ^c^
**rV-*neu*T+CUR**	8	5000 (±0.019)	

^a^ Immune serum titers of BALB-*neu*T-vaccinated mice were determined by ELISA against LTR-Neu after NIH3T3 incubation, using pooled sera (T1) at different dilutions (1:100, 1:500, 1:2500, 1:5000). Serum titer represents the mean value of different serum pools  ±  standard deviation (SD). ^b^ Titer was estimated as the highest immune serum dilution generating a specific absorbance of 1.0 at 492 nm. ^c^ Differences in titers of the sera was evaluated by a two-tailed Student’s *t*-test versus rV-*neu*T+CUR. Neg: negative.

**Table 4:** Effect of rV-*neu*T vaccination on BALB-*neu*T immunoglobulin isotype sera.

	Immunoglobulin Isotype Against Neu					
	IgM	IgG1	IgG2a	IgG2b	IgG3	IgA
**rV-*neu*T**	16.708 ± 1.454 ^a^	17.633 ± 0.621	25.874 ± 0.210	24.534 ± 2.687	9.833 ± 0.058	5.418 ± 1.460
**rV-*neu*T+Corn Oil**	17.158 ± 0.407	16.210 ± 3.706	26.413 ± 0.216	25.511 ± 4.181	9.614 ± 2.103	5.094 ± 1.032
**rV-*neu*T+CUR**	16.372 ± 0.913	15.620 ± 0.439	25.226 ± 3.423	27.700 ± 1.520	9.516 ± 1.165	5.566 ± 0.834

^a^ Results are the mean of the percent (±standard deviation) of each immunoglobulin isotype relative to the total serum immunoglobulin content (at 1:500).

## Supplementary Materials

The following are available online at https://www.mdpi.com/2072-6643/12/5/1417/s1, Figure S1: B cells infiltrating tumors in BALB-*neu*T mice after treatments, Figure S2: Macrophages infiltrating tumors in BALB-*neu*T mice after treatments, Figure S3: Neutrophils infiltrating tumors in BALB-*neu*T mice after treatments.
