# Supplementary material for: Curcumin Enhances the Antitumoral Effect Induced by the Recombinant Vaccinia Neu Vaccine (rV-neuT) in Mice with Transplanted Salivary Gland Carcinoma Cells
Source: Nutrients. 2020 May 14;12(5):1417. doi: 10.3390/nu12051417 (PMC7284625; doi:10.3390/nu12051417)
Supplement: Supplementary file 1 [file nutrients-12-01417-s001.pdf]

Supplementary Materials

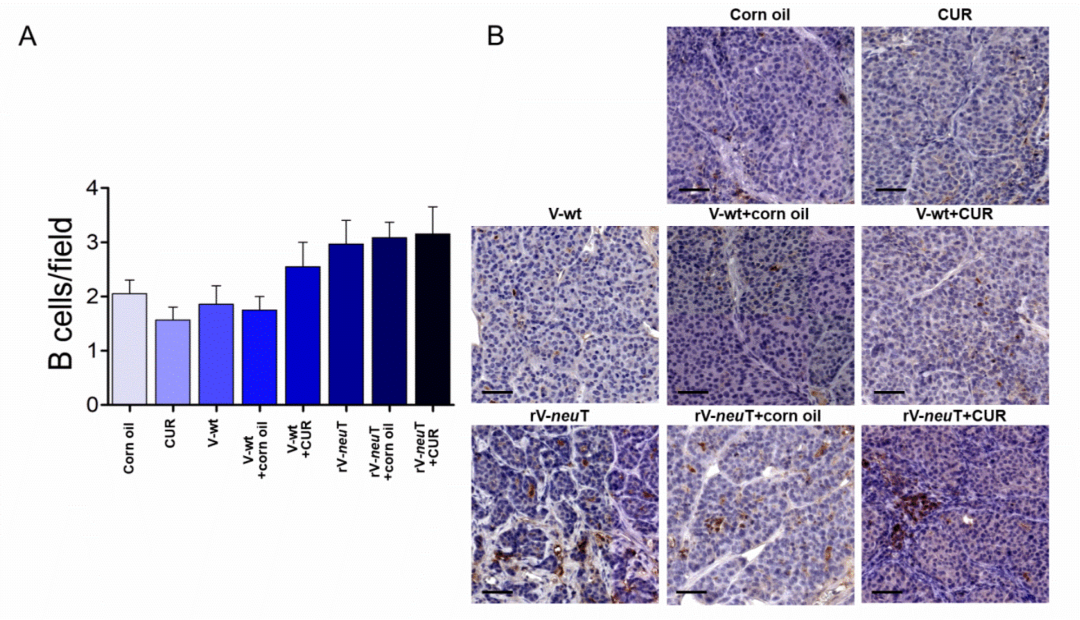

Figure S1. B cells infiltrating tumors in BALB-*neu*T mice after treatments. Tumor tissues from three mice in each group were analysed with IHC for CD19 expression. (**A**) Positive cell count/field averaging 10 representative microscopic field (mean±SD; 1way-ANOVA, Tukey's multiple comparison). (**B**) Representative digital images (20x), scale bar represents 100 µm. CUR: curcumin.

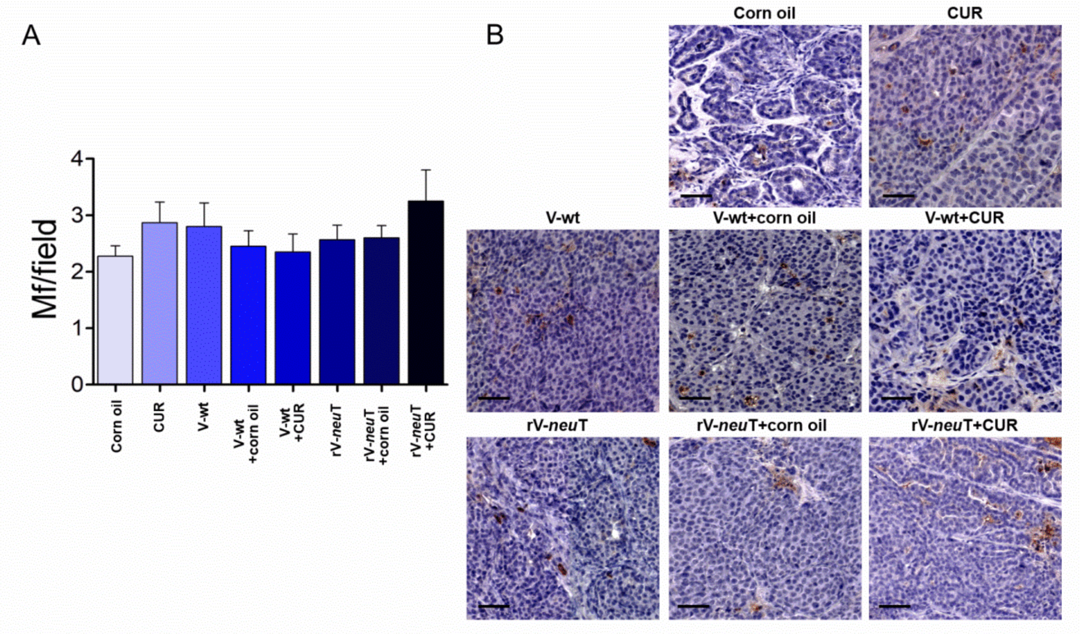

Figure S2. Macrophages infiltrating tumors in BALB-*neu*T mice after treatments. Tumor tissues from three mice in each group were analysed with IHC for f4/80 expression. (**A**) Positive cell count/field averaging 10 representative microscopic field (mean±SD; 1way-ANOVA, Tukey's multiple comparison). (**B**) Representative digital images (20x), scale bar represents 100 µm. Mf: macrophages, CUR: curcumin.

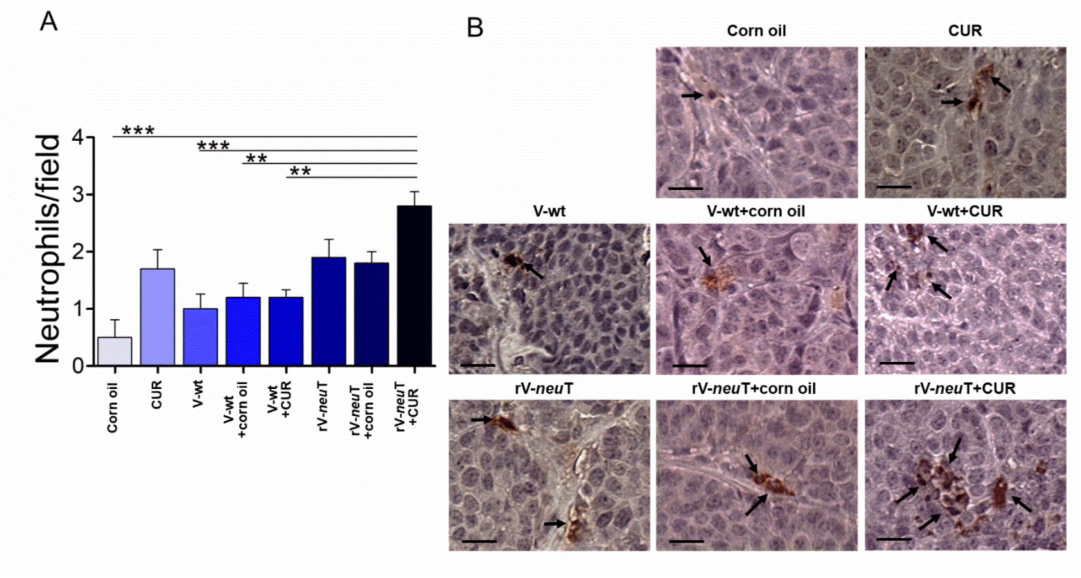

Figure S3. Neutrophils infiltrating tumors in BALB-*neu*T mice after treatments. Tumor tissues from three mice in each group were analysed with IHC for Gr1 expression (arrow). (**A**) Positive cell count/field averaging 10 representative microscopic field (mean±SD, **p≤0.01; ***p≤0.001; 1way-ANOVA, Tukey's multiple comparison). (**B**) Representative digital images (40x), scale bar represents 50 μm. CUR: curcumin.
